# Centrosomal Latency of Incoming Foamy Viruses in Resting Cells

**DOI:** 10.1371/journal.ppat.0030074

**Published:** 2007-05-25

**Authors:** Jacqueline Lehmann-Che, Noémie Renault, Marie Lou Giron, Philippe Roingeard, Emmanuel Clave, Joelle Tobaly-Tapiero, Patricia Bittoun, Antoine Toubert, Hugues de Thé, Ali Saïb

**Affiliations:** 1 Université Paris 7, Paris, France; 2 CNRS, UMR 7151, Paris, France; 3 Université François Rabelais and Centre Hospitalier Régional Universitaire de Tours, Tours, France; 4 INSERM ERI 19, Tours, France; 5 Laboratoire d'Immunologie et d'Histocompatibilité, Assistance Publique–Hôpitaux de Paris, Paris, France; 6 INSERM, U662, Paris, France; Salk Institute for Biological Studies, United States of America

## Abstract

Completion of early stages of retrovirus infection depends on the cell cycle. While gammaretroviruses require mitosis for proviral integration, lentiviruses are able to replicate in post-mitotic non-dividing cells. Resting cells such as naive resting T lymphocytes from peripheral blood cannot be productively infected by retroviruses, including lentiviruses, but the molecular basis of this restriction remains poorly understood. We demonstrate that in G0 resting cells (primary fibroblasts or peripheral T cells), incoming foamy retroviruses accumulate in close proximity to the centrosome, where they lie as structured and assembled capsids for several weeks. Under these settings, virus uncoating is impaired, but upon cell stimulation, Gag proteolysis and capsid disassembly occur, which allows viral infection to proceed. The data imply that foamy virus uncoating is the rate-limiting step for productive infection of primary G0 cells. Incoming foamy retroviruses can stably persist at the centrosome, awaiting cell stimulation to initiate capsid cleavage, nuclear import, and viral gene expression.

## Author Summary

Naive quiescent CD4-positive T cells or monocytes that are in the G0 stage of the cell cycle cannot be productively infected by retroviruses in vitro, but the molecular basis of this restriction remains poorly understood. In this report, we demonstrate that incoming foamy retroviruses remain around the centrosome as structured and assembled capsids for weeks in resting cultures. Under these conditions, virus uncoating is impaired, but upon cell activation, viral capsids undergo proteolysis and disassembly, allowing infection to proceed. Maintenance of incoming viral capsids at the centrosome in resting cells could be a strategy that viruses have evolved to rapidly respond to stimuli received by the cell. The cellular signal triggering the uncoating process upon cell stimulation remains unclear, but is likely linked to the centrosome cycle.

## Introduction

After entry into their host cells, retroviruses undergo uncoating and reverse transcription, leading to the formation of pre-integration complexes, which must then gain access to the nuclear compartment and integrate the provirus into host chromosomes [1]. The cell cycle status is a key determinant for completion of these early stages of infection [2], and retroviruses have been classified based on their ability to productively infect non-cycling cells. Gammaretroviruses, like murine leukemia virus, require mitosis for proviral integration [3], while lentiviruses such as human immunodeficiency virus type 1 (HIV-1) show almost no difference between dividing and non-dividing cells [4]. Indeed, HIV-1 and other lentiviruses can replicate in terminally differentiated and post-mitotic cells such as neurons or macrophages [5,6]. However, like murine leukemia virus, HIV-1 cannot infect naive quiescent CD4-positive T cells or monocytes isolated from peripheral blood that are in the G0 stage of the cell cycle [2,7]. Since reverse transcription does occur in these conditions [8], it is conceivable that other host cell proteins or processes are necessary for the completion of the viral cycle [9].

Foamy viruses (FVs) are complex retroviruses isolated from different animal species, mainly in non-human primates. FVs share with all retroviruses the same organization of the genome, which encodes Gag, Pol, and Env proteins. In addition, the FV genome encodes at least two other proteins, Tas and Bet, which are not incorporated into the viral particle. Remarkably, FVs exhibit some features related to the hepatitis B virus [10]. In particular, they reverse transcribe their RNA genome during the late stages of infection, leading to the presence of infectious viral DNA in extracellular virions [11]. Moreover, the structural FV Gag presents specific characteristics that set it clearly apart from other retroviral Gag proteins. In particular, FV Gag maturation by the viral protease does not lead to the formation of the canonical matrix, capsid, and nucleocapsid products. Rather, the Gag precursor is partially cleaved by the viral protease near its C terminus into a mature product, before or during budding. This results in the presence of two Gag proteins of 71 kDa and 68 kDa in extracellular virions [12]. We have previously reported that upon entry into target cells and prior to nuclear translocation, incoming FV capsids traffic along the microtubule network to reach the microtubule-organizing centre (MTOC) [13,14], which includes the centrosome in animal cells [15]. A similar route has been described for HIV-1 [16]. Drugs disrupting the microtubule network, such as nocodazole or colchicine, largely prevent early intracellular FV trafficking [13], as well as that of HIV-1 [16]. Gag also targets the pericentrosomal region for capsid assembly during the late stages of infection [17]. Similar to murine leukemia virus, productive FV infection requires passage through mitosis [18,19]. Like all other animal retroviruses, FVs do not productively infect cells arrested in the G0 stage of the cell cycle, such as peripheral T lymphocytes or growth-arrested fibroblasts in vitro [19]. To gain insight into this restriction, we have focused our attention on early stages of FV replication in G0 cells.

Here, we demonstrate that incoming FVs stably localize at the vicinity of the MTOC as structured and assembled capsids for several weeks in resting cultures in vitro. Upon cell stimulation, Gag proteolysis and capsid disassembly take place, allowing infection to proceed. Altogether, these data demonstrate that the centrosome represents a cellular site around which incoming viruses persist as a stable pre-integration intermediate in resting cells, and that virus uncoating is the rate-limiting step for FV infection in growth-arrested cells.

## Results

**Figure 1:**
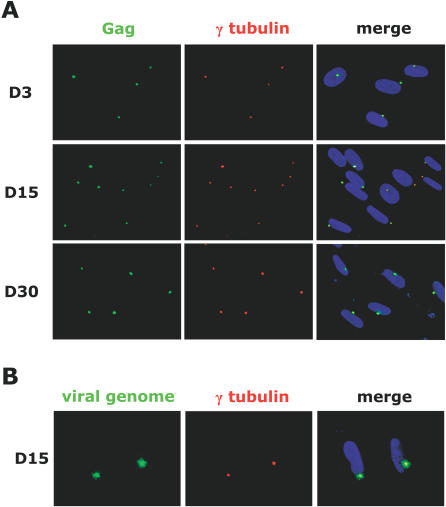
Intracellular Localization of Incoming PFV in Resting MRC5 Cells (A) Sub-cellular localization of incoming Gag proteins studied by confocal microscopy following indirect immunofluorescence at 3, 15, and 30 d p.i. using anti-Gag antiserum (green). At these time points, Gag is exclusively detected at the MTOC, stained with γ-tubulin abs (red). Nuclei are stained with DAPI (blue). (B) Sub-cellular localization of the viral DNA genome from incoming PFV analyzed by confocal microscopy following in situ hybridization 15 d p.i. using a biotin-labeled PFV full-length probe (green). As well as incoming Gag, the viral DNA genome localizes at the MTOC (revealed with anti-γ-tubulin abs, red) 15 d p.i. Nuclei are stained with DAPI (blue).

Cycling and resting human primary MRC5 fibroblastic cells were infected with the prototypic primate foamy virus (PFV) at a multiplicity of infection (m.o.i.) of 1, and cell-free supernatant was collected 48 h later for virus titration. Consistent with previous reports [18,19], we found that PFV does not productively infect G0 resting cells (unpublished data). To investigate the molecular mechanisms involved in the restriction of FV replication cycle in G0 resting cells, we first analyzed the distribution of incoming viral components in resting MRC5 cells. As early as 4 h post-infection (p.i.)., we observed that incoming Gag proteins localized at the centrosome, which was revealed with anti-γ-tubulin antibodies (abs). These findings are consistent with previous observations showing the pericentrosomal concentration of incoming FVs after trafficking along the microtubule network in cycling cells [13]. Remarkably, while in cycling cells, incoming Gag antigens are no longer observed near this organelle 10 h p.i. ([20]; unpublished data); in resting cells, centrosomal localization of viral antigens was consistently observed up to 30 d p.i. (Figure 1A). We then investigated the localization of the viral genome in infected resting cells. By fluorescent in situ hybridization, using the entire PFV DNA genome as a probe, we found that the incoming viral DNA genome also localized at the centrosome in resting MRC5 cells 15 d p.i. (Figure 1B). Therefore, both incoming FV antigens and the viral DNA genome reside at the MTOC of resting cells for several weeks after infection.

**Figure 2:**
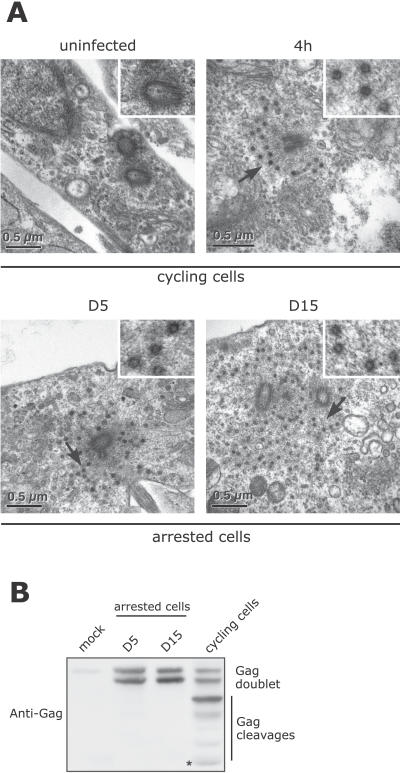
Characterization of Incoming PFV Particles in Resting MRC5 Cells (A) Electron micrographs of uninfected and infected cycling and resting cells. Normally shaped intracellular capsids (pointed out by a black arrow) are observed in PFV-infected cycling cells at 4 h p.i. In resting cells at 5 or 15 d p.i., similar intact capsids (pointed out by a black arrow) are observed surrounding the MTOC. High magnifications are presented in the right corner. No viral capsids are detected in uninfected cells. (B) Western blot analysis of protein extracts from PFV-infected cycling and resting cells using anti-Gag abs. Analysis of protein lysates performed 7 h p.i. in cycling cells reveals the presence of the characteristic Gag doublet (71–68 kDa) and early Gag cleavage products. The 38-kDa Gag product (*) results from the action of the viral protease. In resting cells, 5 or 15 d p.i., these shorter Gag products are absent.

To visualize the status (assembled capsids or not) of incoming viruses in resting cells, MRC5 cells were analyzed by electron microscopy (EM) at different time points after infection. In cycling cells (Figure 2A), incoming FVs were observed at the centrosome 4 h p.i., mainly as structured and assembled capsids, confirming that uncoating is not complete at this time point [20]. At later time points, these viral capsids completely disassembled in cycling cells as already reported [20], and viral capsids were never detected in uninfected cells (Figure 2A). Importantly, assembled and structured viral capsids were observed around the centrosome in resting cells 5 or 15 d p.i. (Figure 2A), strongly suggesting that virus uncoating is impaired under these settings.

FV uncoating requires the enzymatic activity of both viral and cellular proteases, which cleave the major structural components of viral capsids (the 71–68 kDa Gag doublet), into shorter fragments [20]. Among these cleavage products, a 38-kDa Gag-derived product specifically results from the action of the viral protease [20]. To confirm at the biochemical level that FV uncoating is inhibited in resting cells, the status of the Gag polyprotein was determined. For that purpose, resting and cycling MRC5 cells were infected with PFV at an m.o.i. of 1, and intracellular viral proteins were analyzed by Western blot using mouse anti-Gag abs. Figure 2B shows that Gag cleavage products, in particular the 38-kDa fragment, were easily detected in cycling cells. On the contrary, FV Gag was not cleaved following infection of resting MRC5 cells and remained as an intact doublet of 71 and 68 kDa (Figure 2B). These results demonstrate the absence of Gag cleavage in resting cells, reflecting the absence of viral uncoating observed by EM.

**Figure 3:**
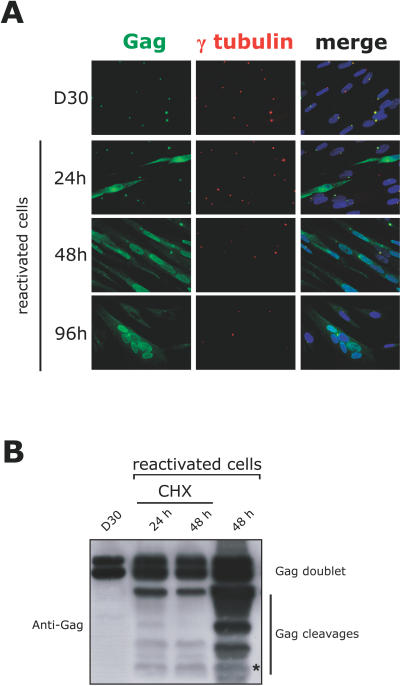
Reactivation of PFV-Infected Resting Cells (A) Sub-cellular localization of Gag proteins (green) studied by confocal microscopy following indirect immunofluorescence using anti-Gag antiserum. Thirty days p.i., Gag proteins mainly localize at the MTOC (stained with anti-γ-tubulin abs in red) in resting cells. In contrast, Gag proteins are detected both in the cytoplasm and the nucleus of these cells at 24, 48, and 96 h post-activation by splitting and serum addition. Syncitia, characteristic of a productive FV infection, appear 96 h post-reactivation. (B) Western blot analysis of protein extracts from resting and activated PFV-infected cells with or without CHX treatment (150 μg/ml). At 30 d p.i. (with m.o.i. of 2), only the Gag doublet at 68 and 71 kDa is detected in resting cells using anti-Gag abs. Under CHX treatment, at 24 h post-reactivation, Gag cleavages are clearly detected, including the 38-kDa Gag product (*) involved in virus uncoating [20].

To assess whether incoming FV capsids at the centrosome of resting cells constitute a stable pre-integration intermediate, which can later be reactivated for productive infection, resting PFV-infected MRC5 cell cultures were stimulated to divide by splitting and serum addition. At different time points following this activation, Gag expression and distribution were analyzed by indirect immunofluorescence using mouse anti-Gag abs. Twenty-four hours after cell activation, we observed that, although Gag still associated to the centrosome, it could be detected in both the cytoplasm and the nucleus of 20% of the cells (Figure 3A). Gag was detected in both compartments in the entire culture 48 h after cell activation, and the formation of numerous syncytia was detected 96 h post-activation (Figure 3A). These results demonstrate that viral replication, which was inhibited in resting cells, resumes upon cell activation. To confirm that cell activation actually triggered virus uncoating, the status of the Gag polyprotein was studied by Western blot. To exclusively analyze incoming viral antigens, avoiding contamination from Gag synthesis and degradation, which might occur following reactivation, these experiments were performed under cycloheximide (CHX) treatment, a translation inhibitor. At 24 h post-reactivation, several Gag cleavage products, notably the 38-kDa fragment [20], were clearly detected in CHX-treated cells (Figure 3B). Moreover, under these settings, accumulation of Gag was observed only in untreated reactivated cells. Altogether, these observations demonstrated that entry into the cell cycle triggered virus uncoating, as assessed by Gag cleavage, and productive infection.

**Figure 4:**
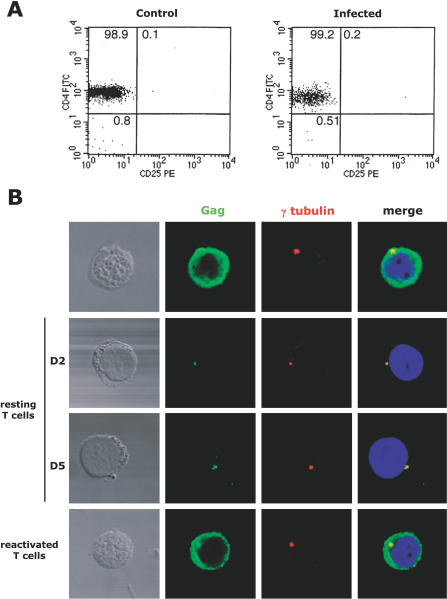
Intracellular Localization of Incoming PFV in Quiescent and Activated CD4-positive T Cells (A) Flow cytometry profile of isolated human resting CD4-positive T cells. Purity of sorted cells is at least 99% and no cell activation is observed 5 d p.i. (B) Sub-cellular localization of Gag proteins studied by confocal microscopy following indirect immunofluorescence using anti-Gag antiserum (green). In activated CD4-positive T cells at 48 h p.i., Gag proteins are not restricted to the MTOC but diffusely localize in the cytoplasm and in the nucleus (top row). In resting CD4-positive T cells at 2 and 5 d p.i., Gag proteins co-localize with γ-tubulin (red), the MTOC marker. Twenty-four hours following cell stimulation of 5 d−infected resting CD4-positive T cells, Gag proteins diffusely localize in the cytoplasm and in the nucleus, similar to what we observed in activated CD4-positive T cells (bottom row).

Several reports have demonstrated that FVs infect lymphocytes in vivo and that infectious particles can be recovered from peripheral T cells of infected animals [21−24]. Therefore, the intracellular distribution of incoming FVs was assessed in one of its natural targets. To this aim, primary resting human CD4-positive T cells were infected with PFV at an m.o.i of 1 and were maintained in a resting state in culture for 5 d without addition of exogenous lymphokines. PFV infection of resting CD4-positive T cells was non-productive (unpublished data) and did not trigger cell activation (Figure 4A). In these cells, localization of incoming capsids was analyzed by confocal microscopy. Consistent with our previous observation of infected resting MRC5 cells, incoming Gag strictly localized at the centrosome from day 2 to day 5 p.i. (Figure 4B). On the contrary, Gag was diffusely distributed in the cytoplasm and the nucleus of activated CD4-positive T cells, indicating that productive replication was taking place in these cells (Figure 4B) [25]. When resting CD4-positive T cells were stimulated to divide 5 d p.i., Gag was no longer observed uniquely at the centrosome but localized diffusely in the cytoplasm and the nucleus at 24 h post-activation (Figure 4B). Taken together, these results confirm that a stable PFV intermediate can persist in the vicinity of the MTOC in primary resting CD4-positive T cells and that T cell activation allows completion of the viral replication cycle.

## Discussion

For all retroviruses, completion of the early steps of the replication cycle depends on the cell cycle status (reviewed in [2,9]). We show that in vitro FV infection is restricted in G0 resting cells, either from fibroblastic (MRC5) or lymphocytic (CD4-positive T cells) origin. We further demonstrate that incoming viral capsids persist in infected G0 resting cells as a stable pre-integration intermediate over a period of at least 30 d in MRC5 cells. Under these conditions, Gag proteolysis, and consequently virus uncoating, is blocked, and incoming viruses are maintained as assembled and structured capsids around the centrosome. Upon cell activation, Gag is cleaved, viral capsids disassemble, and infection proceeds.

Post-mitotic cells such as neurons or macrophages are productively infected by lentiviruses. In contrast, resting G0 cultures in vitro, such as naive T lymphocytes isolated from peripheral blood, cannot be productively infected by any classes of retroviruses, including HIV-1 [8,26−30]. Since reverse transcription is completed in these cells [8,28], additional blocks seem to occur during the early stages of the virus life cycle. Several hypotheses have been raised to elucidate the molecular basis of this restriction. It has been suggested that APOBEC3G, a cellular antiretroviral protein that is associated with the hypermutation of viral DNA through cytidine deamination [31], could inhibit HIV replication as part of a low molecular mass ribonucleoprotein complex in resting T cells. This seems to impair the formation of HIV-1 late reverse transcription products [29]. A recent alternative hypothesis suggests that virus uncoating represents the main rate limiting stage in resting T CD4+ cells, since cellular extracts from activated, but not resting cells, support uncoating of HIV cores in vitro [30,32]. Clearly, our observations suggest this second scenario in the case of FVs. Interestingly, our observations might explain the efficient in vivo transduction of haematopoietic stem cells by FV-derived vectors in mice [33,34]. Indeed, despite the fact that FVs cannot productively infect resting stem cells in vitro, FV vectors can repopulate bone marrow [33−36]. In fact, in vivo implantation of transduced resting stem cells likely triggers their activation, allowing the infection to proceed.

The remarkable stability of the FV intermediate in resting cells that we have evidenced here could be related to the particular mode of replication of these viruses. First, virus uncoating is a relatively late event during FV replication, allowing incoming viral antigens to accumulate in close vicinity to the nuclear compartment as assembled and structured capsids [13,20]. On the contrary, for HIV-1, capsid disassembly in activated and cycling cells seems to start as soon as the viral particles enter into the cytoplasm [16,37]. Second, in contrast to other retroviruses, the presence of an infectious viral DNA genome in incoming capsids could make FV less dependent on the metabolism of the target cell [11]. Our data also demonstrate that the centrosome is a cellular site around which incoming FVs can stably persist, awaiting further cell stimulation for completion of the viral cycle. Recent studies have shown that the centrosome is not a mere spectator of the cell cycle but can exert significant control over it [38]. By providing a scaffold for many cell cycle regulators and their activities [38−40], the centrosome influences cell cycle progression, especially during the transition from G1 to S phase [41,42]. Therefore, this organelle receives and integrates signals from outside the cell and facilitates conversion of these signals into cellular functions. Maintenance of viral capsids at the vicinity of the centrosome in resting cells could be a strategy that some viruses have evolved to rapidly respond to growth stimuli received by the cell. The cellular signal(s) triggering the uncoating process upon cell stimulation remains unclear, but is likely linked to the centrosome cycle.

## Materials and Methods

### Cell cultures.

Human MRC5 fibroblasts were cultured as described [14]. Quiescent MRC5 fibroblasts were generated following confluence, serum starvation, and addition of 10^−6^ M dexamethasone. The cells were cultured in these conditions for 10 d before infection. The cell cycle status was analyzed by pyronine staining as described by [43]. In resting MRC5 cells, less than 3% of cells remain activated, as already reported ([44]; unpublished data). For cell activation, cells were stimulated to divide by subculture and serum addition at different times after infection. CHX treatment was performed at 150 μg/ml immediately after cell activation and maintained during the entire experiment.

Peripheral blood mononuclear cells obtained from healthy donors after informed consent were separated on lymphocytes separation medium (Eurobio, http://www.eurobio.fr). CD4-positive T cells were negatively selected using the CD4-positive T Cell Isolation Kit II (Miltenyi Biotec, http://www.miltenyibiotec.com). Briefly, cells were labeled using a cocktail of biotin-conjugated abs against CD8, CD16, CD19, CD36, CD56, CD123, TCRγδ, and glycophorin A, and anti-biotin magnetic beads. After washing, negative cells were selected by magnetic separation with the autoMACS Separator (Miltenyi Biotec). The cells were then labeled with FITC-anti-CD8 (clone SK1; BD Biosciences, http://www.bdbiosciences.com), anti-CD14 (Clone TUK4, Miltenyi Biotec), Phycoerythrin-conjugated anti-CD25 (clone 4E3, Miltenyi Biotec), and anti HLA-DR (L243, BD Biosciences) ab, washed, and sorted on a FACSVantage. The purity was examined by flow cytometry with the same ab and an allophycocyanin-conjugated anti-CD4 (clone RPA-T4, BD Biosciences). Typically, cells were more than 99% negative for the activation markers (CD25 and HLA-DR) and more than 98% positive for the CD4. Activated CD4-positive T cells were prepared by incubating negatively selected CD4 T cells in RPMI 10% normal human serum and 1 μg/ml PHA-L (Sigma, http://www.sigmaaldrich.com). At day 3, 150 UI/ml of interleukin 2 (IL-2; Promocell, http://www.promocell.com) was added to the cells. Activation status of the control and infected CD4 T cells were checked by flow cytometry with the ab anti-CD4 FITC and an anti-CD25 or HLA DR PE ab.

### PFV infection and titration.

MRC5 cultures or CD4-positive T cells were infected by spinoculation at an m.o.i. of 1 for 1 h 30 min at 30 °C. Titres were determined by infection using FAG cells (BHK cells stably harbouring the GFP gene under the control of the PFVU3 promoter) [45].

### Immunocytochemistry.

MRC5 cells grown on glass coverslips were infected with PFV at an m.o.i. of 1 by spinoculation for 1 h 30 min at 30 °C. After different times p.i., cells were rinsed with PBS, fixed for 10 min at 4 °C with 4% PFA, and permeabilized for 5 min at −20 °C with ice-cold methanol. Cells were incubated successively with mouse polyclonal anti-Gag serum overnight at 4 °C (1/250) and with rabbit polyclonal γ-tubulin antiserum (1/2000; Abcam, http://www.abcam.com) for 1 h at 37 °C. Cells were washed and incubated for 1 h with a 1/500 dilution of appropriate fluorescent-labeled secondary abs. Nuclei were stained with 4'-6-diamidino-2-phenylindole (DAPI) and the coverslips were mounted in Moviol. Confocal microscopy observations were performed with a laser-scanning confocal microscope (LSM510 Meta; Carl Zeiss, http://www.zeiss.com) equipped with an Axiovert 200 M inverted microscope using a Plan Apo 63_/1.4-N oil immersion objective.

### Immunolocalization in combination with fluorescence in situ hybridization.

Fifteen days following PFV infection (m.o.i. of 1), arrested MRC5 cells were fixed with 4% PFA, permeabilized with 0.2% Triton X-100, and incubated with γ-tubulin antiserum (Abcam). Then, cells were fixed a second time in 4% PFA for 20 min at room temperature to cross-link bound abs. Incubation with the secondary ab was performed during the fluorescent in situ hybridization detection step, performed as described [20]. Briefly, cells were treated with RNase at 100 μg/ml in PBS for 30 min at 37 °C and incubated with probe (plasmid p13 containing the entire PFV genome) overnight at 37 °C. Probes were labeled with FITC-avidin DN (1/200; Vector Laboratories, http://www.vectorlabs.com) and signals were amplified with biotinylated anti-avidin D (1/500, Vector Laboratories), followed by another round of FITC-avidin staining. Finally, cells were stained for DNA with DAPI and mounted in Vectashield. Confocal observations were performed as previously described.

### Western blot analysis.

Cell pellets were lysed in Triton buffer (10 mM Tris [pH 7.4]; 50 mM NaCl; 3 mM MgCl2; 1 mM CaCl2; orthovanadate, benzamidine, and protease inhibitor cocktail [Roche, http://www.roche.com] at 1 mM each; 10 mM NaF; and 0.5% Triton X-100) for 30 min at 4 °C and centrifuged for 15 min at 20,000*g*. Resulting pellets were treated with radioimmunoprecipitation buffer (10 mM Tris [pH 7.4]; 150 mM NaCl; orthovanadate, benzamidine, and protease inhibitor cocktail at 1 mM each; 10 mM NaF; 1% deoxycholate; 1% Triton X-100; and 0.1% sodium dodecyl sulfate [SDS]) during an additional 30 min at 4 °C, centrifuged for 15 min at 20,000*g,* collected, and diluted in Laemmli buffer. Samples were migrated on an SDS–10% polyacrylamide gel, and proteins were transferred onto cellulose nitrate membrane (Optitran BA-S83; Schleicher-Schuell, http://www.schleicher-schuell.com), incubated with appropriated abs, and detected by enhanced chemiluminescence (Amersham ECL Advance Western Blotting Detection Kit, http://www.gelifesciences.com).

### Electron microscopy.

For EM studies, MRC5 cells were infected at an m.o.i. of 5 as described above and fixed in situ by incubation for 48 h in 4% PFA and 1% glutaraldehyde in 0.1 M phosphate buffer (pH 7.2); they were then post-fixed by incubation for 1 h with 2% osmium tetroxide (Electron Microscopy Science, http://www.emsdiasum.com). Next, MRC5 cells were dehydrated in a graded ethanol series, cleared in propylene oxyde, and then embedded in Epon resin (Sigma), which was allowed to polymerize for 48 h at 60 °C. Ultrathin sections were cut, stained with 5% uranyl acetate/5% lead citrate, and then placed on EM grids coated with collodion membrane. They were then observed with a Jeol 1010 transmission electron microscope (Jeol, http://www.jeol.com).

## Abbreviations

ab: antibody
CHX: cycloheximide
EM: electron microscopy
FV: foamy virus
HIV-1: human immunodeficiency virus type 1
m.o.i.: multiplicity of infection
MTOC: microtubule-organizing centre
p.i.: post-infection
PFV: primate foamy virus
